# Target-cell-specific short-term plasticity in local circuits

**DOI:** 10.3389/fnsyn.2013.00011

**Published:** 2013-12-06

**Authors:** Arne V. Blackman, Therese Abrahamsson, Rui Ponte Costa, Txomin Lalanne, P. Jesper Sjöström

**Affiliations:** ^1^Department of Neuroscience, Physiology and Pharmacology, University College LondonLondon, UK; ^2^Department of Neurology and Neurosurgery, Centre for Research in Neuroscience, The Research Institute of the McGill University Health Centre, Montreal General HospitalMontreal, QC, Canada; ^3^Neuroinformatics Doctoral Training Centre, School of Informatics, Institute for Adaptive and Neural Computation, University of EdinburghEdinburgh, UK; ^4^Integrated Program in Neuroscience, McGill UniversityMontreal, QC, Canada

**Keywords:** short-term plasticity, synapse specificity, synaptic disease, network models, synapse formation, development

## Abstract

Short-term plasticity (STP) denotes changes in synaptic strength that last up to tens of seconds. It is generally thought that STP impacts information transfer across synaptic connections and may thereby provide neurons with, for example, the ability to detect input coherence, to maintain stability and to promote synchronization. STP is due to a combination of mechanisms, including vesicle depletion and calcium accumulation in synaptic terminals. Different forms of STP exist, depending on many factors, including synapse type. Recent evidence shows that synapse dependence holds true even for connections that originate from a single presynaptic cell, which implies that postsynaptic target cell type can determine synaptic short-term dynamics. This arrangement is surprising, since STP itself is chiefly due to presynaptic mechanisms. Target-specific synaptic dynamics in addition imply that STP is not a bug resulting from synapses fatiguing when driven too hard, but rather a feature that is selectively implemented in the brain for specific functional purposes. As an example, target-specific STP results in sequential somatic and dendritic inhibition in neocortical and hippocampal excitatory cells during high-frequency firing. Recent studies also show that the Elfn1 gene specifically controls STP at some synapse types. In addition, presynaptic NMDA receptors have been implicated in synapse-specific control of synaptic dynamics during high-frequency activity. We argue that synapse-specific STP deserves considerable further study, both experimentally and theoretically, since its function is not well known. We propose that synapse-specific STP has to be understood in the context of the local circuit, which requires combining different scientific disciplines ranging from molecular biology through electrophysiology to computer modeling.

## Introduction

The functioning of the brain is governed by its neuronal connectivity and by the synaptic dynamics of these connections. Learning and information storage in the brain, for example, are widely thought to be due to long-term changes in connective strength, as postulated by Donald Hebb (1949) and others before him (Markram et al., 2011). Such changes last for hours, days, and weeks. But there are also other forms of synaptic plasticity that are active on considerably faster time scales, such as short-term depression and facilitation, and these last from a few milliseconds to tens of seconds (Zucker and Regehr, 2002; Abbott and Regehr, 2004).

**Figure 1:**
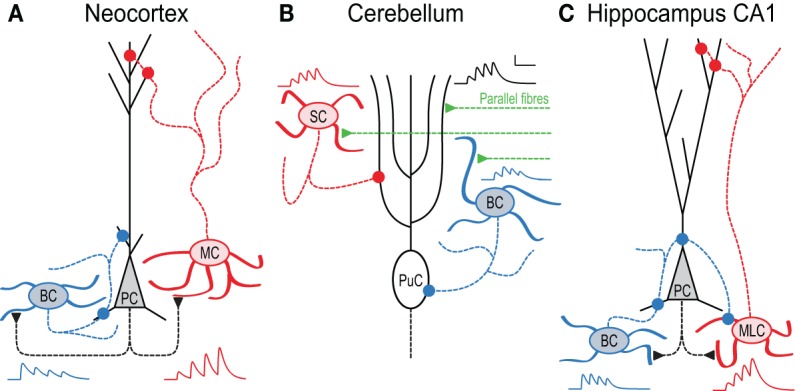
**In local circuits, target-cell specific STP remaps spiking across the somato-dendritic axis. (A)** Pyramidal cell (PC) inputs to basket cells (BC) are short-term depressing, whereas those to Martinotti cells (MC) are facilitating (Markram et al., 1998). As a result, high-frequency PC firing (Larkum et al., 1999; Murayama et al., 2009) activates MCs later than BCs, an effect that is amplified by presynaptic NMDA receptors (Figure 3 and Buchanan et al., 2012). BCs in turn innervate PCs perisomatically (Buchanan et al., 2012), whereas MCs contact the apical dendrite (Silberberg and Markram, 2007). **(B)** In cerebellum, synapses between parallel fibers (PF) and Purkinje cells (PuC) facilitate, as do connections to stellate cells (SC). In contrast, PF synapses onto BCs depress, so high-frequency PF activity triggers SCs later than BCs, leading to early onset somatic and late-onset dendritic PuC inhibition (Bao et al., 2010). **(C)** In hippocampus CA1, PCs connect to two distinct *stratum oriens* IN types with contrasting STP. Onset-transient BCs receive depressing input and target PCs and other INs perisomatically, whilst late-transient Martinotti-like cells (MLC) receive facilitating input and target dendrites (although see Hefft and Jonas, 2005; Glickfeld and Scanziani, 2006). During 50 Hz firing therefore, inhibition of PCs shifts from somatic to dendritic (Pouille and Scanziani, 2004). Early-onset BC inhibition of MLCs may additionally amplify this effect (Lovett-Barron et al., 2012). All synaptic traces were simulated based on data in (Pouille and Scanziani, 2004; Bao et al., 2010; Buchanan et al., 2012).

Synaptic short-term plasticity (STP) is thought to result from a combination of mechanisms, chiefly presynaptic ones, including vesicle depletion and accumulation of calcium in the presynaptic terminal during prolonged high-frequency activity, but desensitization of postsynaptic neurotransmitter receptors also matter (Zucker and Regehr, 2002; Thomson, 2003; Abbott and Regehr, 2004; Fioravante and Regehr, 2011). STP is not an epiphenomenal synaptic defect due to fatigue during high-frequency activity, but rather a feature that the brain relies on to process information and to maintain the balance of excitation and inhibition. Indeed, STP depends specifically on factors such as developmental age (Pouzat and Hestrin, 1997; Reyes and Sakmann, 1999; Cheetham and Fox, 2010), neocortical layer (Reyes and Sakmann, 1999), brain area (Wang et al., 2006; Cheetham and Fox, 2010), postsynaptic cell-type (Markram et al., 1998; Beierlein et al., 2003; Buchanan et al., 2012), and sensory experience (Finnerty et al., 1999; Cheetham and Fox, 2011; Liu et al., 2012). Differences in STP have been identified at connections between two neocortical pyramidal cells (PCs) and those from PCs to various interneurons (INs) (Thomson et al., 1996). For example, connections from PCs to basket cells (BCs) typically exhibit short-term depression, whereas those from PCs to Martinotti cells (MCs) show striking facilitation (Figure 1A) (Markram et al., 1998; Reyes et al., 1998; Buchanan et al., 2012). Because the type of STP that is active at a given synapse type critically determines the type of information it transfers, the same presynaptic cell may thus transmit quite different information to different classes of postsynaptic cells (Markram et al., 1998). As an illustration, short-term depressing and facilitating synapses optimally transfer information at low and high frequencies, respectively (Fuhrmann et al., 2002). Short-term depression also emphasizes temporal coherence of inputs at the expense of rate coding, and these different synaptic dynamics may result in quite distinct regular or irregular activity regimes in recurrently connected networks (Tsodyks and Markram, 1997; Tsodyks et al., 1998).

Here, we overview target-cell-specific STP in local circuits and discuss its potential mechanisms as well as its functional implications.

### Functional implications of STP

One of the most obvious functional implications of STP is that it filters information flow across the synapse (Dittman et al., 2000; Fortune and Rose, 2001; Fuhrmann et al., 2002). For example, a facilitating connection, e.g., from a PC to an MC (Figure 1A), requires several spikes to elicit suprathreshold responses in the MC. A PC-MC connection thus constitutes a high-pass filter, since only high-frequency trains elicit postsynaptic spikes. Conversely, a short-term depressing synapse—such as that between a PC and a BC (Figure 1A)—may trigger spikes in the recipient cell after the first few spikes, after which it needs to recover to the initial high-release state. Low frequencies are therefore more efficacious than high frequencies at bringing the postsynaptic cell to threshold, which corresponds to a low-pass filter. Intermediate forms of plasticity may elicit band-pass filtering.

Because they act as high-pass filters, facilitating synapses may function as burst detectors (Maass and Zador, 1999; Matveev and Wang, 2000). Lisman (1997) proposed that bursts are particularly important for information coding in several brain regions. Bursts are more reliable at carrying information than single spikes, which is useful as individual spikes may result from noise. Indeed, bursts are particularly reliable triggers of long-term plasticity and of information storage (e.g., Pike et al., 1999; Nevian and Sakmann, 2006). The hippocampal CA1 region in particular may have STP tuned for burst detection (Klyachko and Stevens, 2006).

However, bursting may also limit long-term plasticity. In neocortex, bursting PCs recruit MCs (Murayama et al., 2009) because of the strongly facilitating PC-MC connection (Figure 1A). MCs in turn form inhibitory synapses on the distal apical dendrite of PCs that are well suited to self-limit high-frequency excitatory firing in neocortex (Silberberg and Markram, 2007; Berger et al., 2009, 2010). Because Hebbian plasticity of excitatory inputs onto the distal apical dendrite of layer-5 PCs (Figure 1A) requires dendritic depolarization (Sjöström and Häusser, 2006), high-frequency firing may thus via MCs reduce potentiation.

Just like facilitation enables burst detection, short-term depression can help decorrelate and regularize activity (Goldman et al., 1999) (although it can also promote synchrony in some cases, see Tsodyks et al., 2000). In general, short-term depression of excitatory inputs is likely to help stabilize activity in local circuits, by rendering neurons sensitive to changes in input frequency rather than to the absolute rates (Abbott et al., 1997; Tsodyks and Markram, 1997). STP may thus provide neuronal circuits with a degree of self-stabilization, by ensuring that synaptic drive rapidly dies off during high-frequency activation. In addition, excitatory synapses may depress faster than some inhibitory connections do, thus shifting the balance of excitation and inhibition in favor of the latter during high-frequency firing (Galarreta and Hestrin, 1998; Varela et al., 1999). However, connections from neocortical PCs to BCs tend to short-term depress slightly faster than those between PCs (Costa et al., 2013). Nevertheless, STP generally helps restrict activity levels, even at relatively low rates (Sussillo et al., 2007). Differences in STP at PC-PC and PC-IN connections result in different cell types being maximally activated at different times, an effect that is context-dependent. Cross-correlations between PCs and facilitating INs show greater peak lag values than those between PCs, or to depressing INs; an effect that is dependent on patterned presynaptic activity (Silberberg et al., 2004). Evidence suggests that PV and SOM INs are involved with normalizing, divisive inhibition and subtractive, response selectivity sharpening inhibition, respectively (Wilson et al., 2012). As inputs to these cell types exhibit differing STP, this may control the relative timing of these functions during network activity.

Conversely, facilitation may help sustain activity, which is essential for the proper functioning of several neuronal circuit types. In working memory tasks, critical information is temporarily held in the form of persistent activity in prefrontal cortex while awaiting a relevant cue (Goldman-Rakic, 1995). Although the precise mechanisms of working memory circuits remain unclear, prefrontal cortex PCs interconnect with synapses that are more facilitating than in other cortical regions (Wang et al., 2006). Computer modeling revealed that facilitating synapses might sustain persistent activity in working memory (Mongillo et al., 2008; Hansel and Mato, 2013). Increased synaptic augmentation in prefrontal circuits has also been implicated (Varela et al., 1997; Hempel et al., 2000). In agreement, disruption of the gene Dgcr8, which affects STP in prefrontal cortex layer-5 PCs, also impacts working memory performance (Fenelon et al., 2011; Arguello and Gogos, 2012).

STP can also help explain adaptation. For example, stimulus-evoked suprathreshold responses in barrel cortex quickly reduce to subthreshold levels when repeated, which has been directly linked to short-term depression of thalamocortical afferents (Chung et al., 2002). In visual cortex, short-term depression explains contrast adaptation (Chance et al., 1998), and input-specific short-term depression explains stimulus-specific adaptation (Chance and Abbott, 2001). Differences in STP may also explain differential sensitivity of auditory pathways to timing and intensity (MacLeod, 2011). Differential target-specific changes in STP at thalamic inputs to two cortical IN types may serve to compensate for reduced sound-driven activity in animals with developmental sensorineural hearing loss (Takesian et al., 2013).

## STP is target cell specific

Early evidence from crustacean muscle fibers suggested that terminals originating from the same motor axon might exhibit facilitation or depression depending on postsynaptic target (Atwood, 1967; Atwood and Bittner, 1971). Because target-cell-specific synaptic transmission could be essential for controlling functionally distinct components of neuronal circuits, several studies have since focused on this issue in the mammalian central nervous system (Toth and McBain, 2000; Thomson, 2003; Pelkey and McBain, 2007).

Much evidence for target-specific STP has been found in neocortex. PCs typically interconnect with depressing synapses, whilst PC connections to some IN types facilitate (Markram et al., 1998; Gupta et al., 2000). In layer 2/3, PCs form facilitating synapses with bitufted INs expressing somatostatin (SOM), but establish depressing synapses onto multipolar INs expressing parvalbumin (PV) (Reyes et al., 1998; Rozov et al., 2001). Presynaptic bouton calcium signals at these connections also depend on the target cell: connections to bitufted INs exhibit small calcium signals, whilst connections to multipolar INs show three times larger calcium signals (Koester and Johnston, 2005). Optical quantal analysis also suggests that *p*_release_ is specific to the target cell, with different synaptic contacts of the same connection exhibiting similar release probabilities (Koester and Johnston, 2005). In layer 5, similar differences in STP are seen as in layer 2/3, with depressing PC-PC, facilitating PC-SOM IN and depressing PC-PV IN synapses (see Figure 1A, below and Buchanan et al., 2012). As SOM-positive INs typically target PC apical dendrites, whilst PV-positive INs are often perisomatic-targeting BCs (Markram et al., 2004), the differences in STP seen from PCs to these cell types can result in inhibition hyperpolarising PC somata and dendrites in sequence during high-frequency firing (Figure 1A and Buchanan et al., 2012).

In the cerebellum, target-specific STP can be seen at parallel fiber (PF) connections from granule cells to Purkinje cells and basket or stellate INs, with similar functional consequences. Whilst PF synapses onto Purkinje or stellate cells exhibit persistent facilitation, PF inputs to BCs display short-term depression following initial paired-pulse facilitation (Bao et al., 2010). As seen onto Golgi cells, deletion of the presynaptic protein Munc13 increases facilitation at PF-BC synapses (Beierlein et al., 2007; Bao et al., 2010). This target-specific STP suggests specific functionality, since BCs target Purkinje cells perisomatically, whereas stellate cells innervate their dendrites (Figure 1B). As inputs to BCs short-term depress while those to stellate cells facilitate, high-frequency granule cell firing (Chadderton et al., 2004) recruits BCs first, with stellate cells lagging. As in neocortex, PFs may thus hyperpolarize Purkinje cell soma and dendrites sequentially (Bao et al., 2010) (see below and Pouille and Scanziani, 2004).

Within hippocampal CA1, PCs contact PV-positive SO INs with depressing, high *p*_release_ connections, and different INs with facilitating, low *p*_release_ synapses (Thomson, 1997; Ali et al., 1998; Ali and Thomson, 1998). In Figure 1C, we call these latter INs Martinotti-like cells (MLCs), although they may in actuality be a combination of SOM and CCK positive INs (Hefft and Jonas, 2005; Glickfeld and Scanziani, 2006), thus potentially making the hippocampal scenario more complex than the neocortical one (Figure 1A). Regardless, during high frequency spiking in CA1 PCs, this leads to early-onset firing in some INs and late onset in others. Because the former innervate PCs perisomatically whereas the latter predominantly target the apical dendrite (Figure 1C), this leads to temporal information being remapped spatially across the somato-dendritic axis (Pouille and Scanziani, 2004), as seen in neocortex and cerebellum. Additionally, early-onset INs may also inhibit late-onset INs (Lovett-Barron et al., 2012), accentuating further the temporal difference in firing.

**Figure 2:**
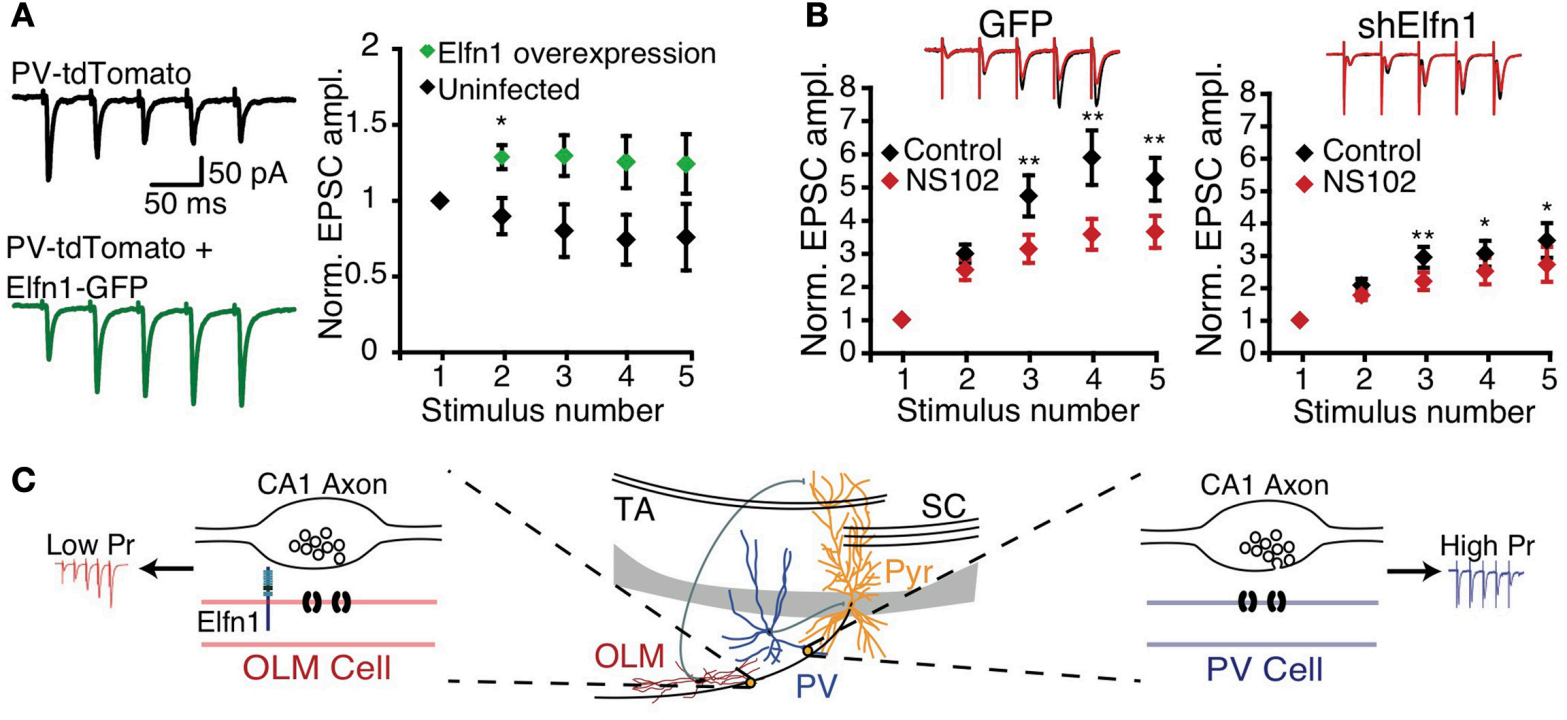
**Postsynaptic Elfn1 controls presynaptic transmitter release**. **(A)** Five stimuli at 20 Hz in the *alveus* produce short-term depression in *stratum pyramidale* or *oriens* PV INs (black). PV neurons overexpressing Elfn1-GFP no longer short-term depress (green). Left: example traces, right: ensemble normalized EPSC amplitude. **(B)** SOM INs normally facilitate (black), but facilitation is attenuated by GluR6-selective kainate receptor antagonist NS102 (left, red, *n* = 8). ShElfn1 expression reduces NS102 effect (right, *n* = 14). Top: example responses, bottom: ensemble normalized EPSC amplitude. **(C)** Proposed Elfn1 mechanism: CA1 PCs contact PV (blue) and SOM (red) INs (middle). PC synapses onto PV neurons lacking Elfn1 have short-term depression (blue). However, SOM INs expressing Elfn1 transsynaptically reduce probability of release, leading to facilitating PC-SOM IN connections (red). From (Sylwestrak and Ghosh, 2012). Reprinted with permission from AAAS. **(A)** ^*^P < 0.05 by Mann-Whitney U test. **(B)** ^*^P < 0.05; ^**^P < 0.01 by ANOVA with Tukey's post-hoc test.

These findings suggest that postsynaptic cell type can determine presynaptic terminal properties. Sylwestrak and Ghosh (2012) recently identified a candidate transsynaptic regulator gene, Elfn1, which was expressed preferentially in SOM-positive *oriens lacunosum-moleculare* (OLM) INs and that may signal postsynaptic identity to presynaptic terminals. Knockdown of Elfn1 in OLM INs led to a marked reduction in facilitation and in an increase of *p*_release_ at connections from CA1 PCs, an effect not seen in uninfected neighbor cells (Figure 2). Conversely, overexpression of Elfn1 in PV INs converted its short-term depressing CA1 PC inputs to facilitation (Figure 2) (Sylwestrak and Ghosh, 2012), suggesting that Elfn1 controls whether a synapse is facilitating or depressing. Mechanistically, Elfn1 may promote facilitation via synapse-specific presynaptic GluR6 expression, because GluR6 kainate receptor blockade reduced facilitation at PC to OLM IN synapses, but less so at connections to Elfn1 knockdown neurons (Figure 2) (Sylwestrak and Ghosh, 2012).

Other hippocampal connections show more varied target-specific STP. For example, synapses from CA3 PCs to CA1 *stratum oriens* (SO) INs exhibit greater facilitation than those to CA1 PCs (Hampson et al., 1998). Additionally, CA3 PC terminals contacting mGluR1a-positive SO INs express high levels of mGluR7 (Shigemoto et al., 1996) that selectively decrease transmitter release at these synapses (Scanziani et al., 1998). In contrast, (Sun et al., 2005) observed that connections from CA3 PCs to *stratum radiatum* (SR) INs had less facilitation or even depression. Presynaptic terminal properties can thus differ widely depending on the target cell, even across IN types. Properly determining IN class is therefore vital when investigating target-specific STP (Ascoli et al., 2008; DeFelipe et al., 2013).

In CA3, large boutons of mossy fibers originating from granule cells in the dentate gyrus contact PCs, whilst smaller synapses impinge on INs (Acsady et al., 1998). Synapses onto CA3 PCs have many release sites with low *p*_release_, resulting in facilitation. Synapses onto CA3 INs, however, are less facilitating, or can be depressing (Salin et al., 1996; Toth et al., 2000). The balance of excitation and inhibition furthermore depends on frequency: excitatory drive of INs depresses more rapidly at high frequencies, while that onto PCs does not (Mori et al., 2004). Dentate gyrus granule cells firing at low frequencies thus prioritises inhibition, whilst higher frequencies excite postsynaptic PCs. Together, these features may explain high-frequency burst firing in CA3 PCs as an animal enters a place field (Leutgeb et al., 2007). Facilitation at mossy fiber to CA3 PC synapses may additionally rely on presynaptic kainate receptors (Darstein et al., 2003; Scott et al., 2008; Ruiz and Kullmann, 2012) that cause facilitation of presynaptic calcium entry.

Whilst the studies above often describe STP between excitatory cells, target-specificity has usually been established only by comparison to connections onto inhibitory interneurons. Target-specific STP at different excitatory-excitatory connections is therefore an interesting possibility that has been investigated in less detail, perhaps as the recordings required for direct comparison are more difficult to achieve experimentally. This said, some studies have touched on this topic. In neocortex, stellate cells in layer 4 connect with depressing synapses (Egger et al., 1999), similarly to synapses between L4 and L2/3 (Brasier and Feldman, 2008). Hippocampal mossy fiber synapses onto mossy cells in the dentate gyrus exhibit facilitation, as do mossy fiber-CA3 PC synapses (Lysetskiy et al., 2005). Cerebellar mossy fibers synapse on both deep cerebellar nuclei and granule cells with short-term depressing connections (Saviane and Silver, 2006; Zhang and Linden, 2006). Whilst the evidence above seems to suggest less difference between STP onto excitatory cells, there is still the possibility of more subtle differences, similar to those seen between PC-PC and PC-BC connections in visual cortex (Costa et al., 2013). Revealing subtle differences at connections between excitatory neurons may thus require more refined experimental and theoretical techniques (Costa et al., 2013).

## Candidate molecules for target-cell-specific retrograde signalling

Target-cell-specific STP predicts that neighboring presynaptic compartments may possess dissimilar release properties, as governed by their postsynaptic partners (see Figure 1). This scenario requires that the postsynaptic cell can regulate specifically its own presynaptic compartment by retrograde signaling without affecting neighboring presynaptic compartments that synapse onto other cell types (Sylwestrak and Ghosh, 2012). Once a synapse has formed, diffusible retrograde messengers such as endocannabinoids and nitric oxide (Kreitzer and Regehr, 2002; Regehr et al., 2009) thus do not appear to be the most parsimonious players in target-cell-dependent synaptic dynamics, since these render micrometer-scale synapse specificity difficult to achieve. However, several synaptic adhesion molecules enable specific retrograde regulation of presynaptic compartments (see Table 1). Although their involvement in target-cell-specific retrograde signaling *per se* remains to be shown, they constitute appealing candidates.

**Table 1:** **Postsynaptic molecules governing presynaptic transmitter release**.

**Brain region**	**Protein**	**P_rel_**	**P_ves_**	**RRP**	**References**
Hippocampus^*^	N-cadherin	+	?	+	Bozdagi et al., 2004
Embryonic stem cells	N-cadherin	+	X	+	Jungling et al., 2006
Hippocampus^*^	N-cadherin	+	X	+	Vitureira et al., 2012
Brain stem^#^	Neuroligin	+	?	?	Varoqueaux et al., 2006
Hippocampus^*^	Neuroligin	+	?	+	Wittenmayer et al., 2009
Hippocampus¤	PSD95, neuroligin	+	+	?	Futai et al., 2007
Embryonic stem cells/hippocampus¤	Neuroligin/N-cadherin	+	?	+	Stan et al., 2010
Hippocampus^*^	SynCAM	+	?	?	Sara et al., 2005
Hippocampus^*^	SAP97	+	?	+	Regalado et al., 2006
Hippocampus^*^	PSD95	+	?	?	El-Husseini et al., 2000
Hippocampus^*^	SHANK1	+	?	+	Sala et al., 2001

P_rel_, release probability; P_ves_, vesicular release probability; RRP, readily releasable pool;

* dissociated cell culture;

¤, organotypic slices;

# acute slices;

*+, the protein has positive effect; X, the protein has no effect; ?, effect of the protein was not investigated*.

**Cadherins** are a group of synaptic adhesion molecules widely believed to be involved in e.g., synapse formation (Takeichi and Abe, 2005; Takeichi, 2007). To establish transsynaptic interactions, postsynaptic cadherins bind to the extracellular domain of the same type of cadherins located in the presynaptic terminal. These molecules appear to have an important role in mediating synaptic plasticity and to control *p*_release_. In a study by Bozdagi et al. (2004), postsynaptic blockade of N-cadherin, a cadherin molecule expressed at excitatory synapses, lead to smaller synapses, decreased vesicle recycling, and lowered *p*_release_. Another study, in this case using presynaptic wild-type neocortical neurons paired with embryonic stem cells lacking N-cadherin, showed enhanced short-term depression in response to 50-Hz stimulation (Jungling et al., 2006). In some stimulation conditions, however, absence of N-cadherin surprisingly converted short-term depression to facilitation. The amplitude of the first response of a train was not altered, indicating that the initial release probability was unaffected. This implies a deficiency in maintaining transmitter release probability, causing a synaptic depression. The authors concluded that the reduced release probability was caused by an altered rate of recruitment to the readily releasable vesicle pool. In a more recent study, N-cadherin deletion reduced the total number of vesicles as well as the number of docked vesicles, thereby reducing p_release_ (Vitureira et al., 2012). However, no change in calcium sensitivity was seen. The study also shows that the GluA2 subunit can act as a possible mediator of the effect of N-cadherins. Taken together, these three studies suggest a key role for N-cadherins in governing the dynamics of glutamatergic neurotransmission.

**Neuroligins**, another group of postsynaptic cell adhesion proteins with a role in synapse maturation, has also been implicated in transsynaptic regulation of neurotransmitter release (Dean and Dresbach, 2006). Neuroligins bind to several proteins in the postsynaptic structure such as PSD-95, but also to neurexins, which are membrane surface proteins located in the presynaptic terminal. Neurexins recruit a series of proteins involved in the presynaptic release machinery. Neuroligins can thus via neurexins interact with presynaptic calcium channels, synaptic vesicles, and other release-related proteins (Dean and Dresbach, 2006). In one study, deletion of neuroligins reduced the frequency of both excitatory and inhibitory spontaneous release in respiratory neurons of the brain stem as a direct consequence of reduced *p*_release_ (Varoqueaux et al., 2006), showing that neuroligins are essential for proper presynaptic function. Neuroligins were not, however, necessary for synapse numbers, although synaptic maturation was perturbed (Varoqueaux et al., 2006). Another study showed that the recycling vesicle pool size as well as the frequency of spontaneous excitatory release increased when neuroligin 1 was overexpressed, while its deletion resulted in immature presynaptic terminals and a diminished vesicle pool size (Wittenmayer et al., 2009). Neuroligins bind to **PSD95** postsynaptically, which has previously been shown to accelerate synaptic maturation (El-Husseini et al., 2000). To explore this interaction further, Futai et al. (2007) studied its potential role in regulating *p*_release_. Overexpression of PSD95 or neuroligins reduced paired-pulse ratio, while deletion of the same proteins increased paired-pulse ratio, indicating that either PSD95 or neuroligins can increase *p*_release_. The effect of up-regulating one of the proteins was occluded by down-regulating the other. Overexpression resulted in increased sensitivity to extracellular calcium concentration and in higher glutamate concentration in the synaptic cleft, indicating that more vesicles were released. The effect on release was mediated by presynaptic β-neurexins (Futai et al., 2007). To conclude, neuroligins are well suited for regulating *p*_release_, possibly by controlling both the vesicle pool size and the calcium sensitivity. In addition to their individual effects on transmitter release, the cadherin and neuroligin systems can cooperate in regulating release from presynaptic terminals. Stan et al. (2010) found that N-cadherin caused neuroligin to accumulate postsynaptically and that it also activated neuroligin via S-SCAM, a scaffolding molecule, which in turn led to clustering of presynaptic vesicles. Hence, N-cadherin is required for neuroligin to increase *p*_release_.

There are several other molecules that have been suggested to play a part in transsynaptic regulation of presynaptic transmitter release via activation of either the neuroligin or the cadherin system. One example is **SynCAM**, an immunoglobulin domain–containing homophilic synaptic cell adhesion molecule, that when overexpressed in hippocampal neurons brings about an increase in excitatory spontaneous release (Biederer et al., 2002; Sara et al., 2005). Likewise, overexpression of **SAP97**, a postsynaptic scaffolding protein, increased vesicle release probability, presynaptic protein content, and the size of the active zone (Regalado et al., 2006). Overexpression of **Shank1**, a synaptic scaffolding protein implicated in autism (Jiang and Ehlers, 2013), has been shown to enhance spontaneous excitatory release and the vesicle pool size (Sala et al., 2001).

## Target-specific control of transmitter release by presynaptic ionotropic receptors

The above synaptic adhesion molecules may enable cells to signal specifically to their own presynaptic boutons. The regulation of neurotransmitter release could subsequently be achieved via many different presynaptic mechanisms (reviewed in Zucker and Regehr, 2002; Thomson, 2003; Fioravante and Regehr, 2011), e.g., by controlling the calcium buffer calbindin-D28k (Blatow et al., 2003) or vesicle-priming RIM proteins (Deng et al., 2011a; Han et al., 2011).

In the past decade, attention has turned to presynaptic ionotropic receptors in synapse-specific control of transmitter release (Engelman and MacDermott, 2004). Presynaptic kainate receptors, for example, act as autoreceptors to promote facilitation at Schaffer collateral synapses to CA1 INs but not to CA1 PCs (Sun and Dobrunz, 2006). During high-frequency firing, cerebellar PF synapses onto stellate INs are depressed by presynaptic kainate autoreceptors, while synapses to Purkinje cells are enhanced (Delaney and Jahr, 2002). Probability of release at connections between molecular-layer INs, on the other hand, is increased by calcium-permeable AMPA receptors located in axonal compartments, which are presumably activated by glutamate spillover from nearby PFs (Rossi et al., 2008). Interestingly, no such effect can be observed at connections from molecular-layer INs and Purkinje cells (Rossi et al., 2008), again an example of how the mechanisms that govern synaptic release properties can be determined by the postsynaptic target cell type.

NMDA receptors are tetrameric ionotropic glutamate receptors that have been implicated in memory formation and in disease states such as pain, neurodegeneration and schizophrenia (Paoletti et al., 2013). Interestingly, presynaptically located NMDA receptors (preNMDARs) upregulate neurotransmitter release in visual cortex (Sjöström et al., 2003) and entorhinal cortex (Berretta and Jones, 1996). However, the very existence of preNMDARs has been highly debated (see Duguid and Sjöström, 2006; Corlew et al., 2008; Duguid, 2012). Several recent studies suggest that preNMDARs are only expressed at certain synapse types, which may help explain the controversy. Brasier and Feldman (2008) showed that neocortical preNMDARs were present at excitatory connections from layer 4 to layer 2/3 enhanced neurotransmission, while they were absent at excitatory connections within those layers. Such synapse specificity may also be present in the cerebellum, where only a subset of excised molecular layer IN axon terminals were found to express NMDARs (Fiszman et al., 2005), an observation supported by laser uncaging at axon terminals resulting in NMDA-mediated currents at only 30% of locations (Rossi et al., 2012). If the expression of preNMDARs is synapse specific, what role do these receptors play? Our recent results have shed some light on this (Buchanan et al., 2012).

**Figure 3:**
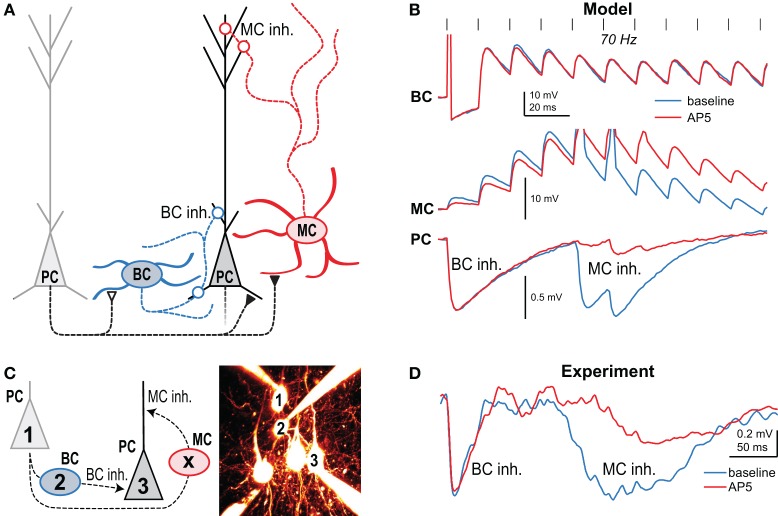
**Presynaptic NMDARs reroute spiking in neocortical microcircuits**. **(A)** Presynaptic NMDARs are specifically expressed at PC-PC and PC-MC synapses (closed symbols), but not at the other connections (open symbols). Circles denote inhibitory synapses. **(B)** A network model with tuned synaptic dynamics predicted that during 70-Hz presynaptic PC firing (vertical lines) blockade of preNMDARs would affect late MC but not early BC inhibition. Traces indicate the model's prediction of synaptic dynamics before (blue) and after (red) NMDAR blockade with AP5. **(C)** In this experiment, 70-Hz spiking in cell 1 resulted in both MC and BC-mediated inhibition in cell 3 when intermediate BC 2 was subthreshold depolarized. Intermediate MC (X) was not patched. **(D)** As predicted, AP5 wash-in affected the amplitude and latency of MC but not BC inhibition, indicating that preNMDARs at PC-MC but not PC-BC synapses boost neurotransmission during high-frequency firing. Reproduced from Buchanan et al. (2012) with permission from Elsevier.

Using NMDA uncaging onto PC axons in combination with paired recordings and pharmacology, we found that in layer 5 of developing visual cortex, preNMDARs are present at connections from PCs to other PCs as well as to MCs, but not to BCs (Figure 3A) (Buchanan et al., 2012). These preNMDARs up-regulate *p*_release_ during high-frequency firing, such as the layer-5 PC complex spike (Larkum et al., 1999; Murayama et al., 2009). A neuronal network computer model tuned to STP data and intrinsic firing properties predicted that during high frequency bursting in PCs, preNMDARs specifically upregulate disynaptic inhibition mediated by MCs but not BCs (Figure 3B) (Buchanan et al., 2012). Model predictions were verified by experiments showing that preNMDAR blockade reduced MC but not BC inhibition (Figures 3C,D) (Buchanan et al., 2012). In conclusion, synapse-specific preNMDAR expression combines with target-cell-specific STP to remap high-frequency spiking along the somato-dendritic axis of PCs. Because MCs inhibit PC dendrites (Figure 1A), preNMDARs strongly impact both PC spiking output (Larkum et al., 1999; Murayama et al., 2009) and plasticity (Sjöström et al., 2008). Since neocortical preNMDARs are downregulated at the end of the critical period (Corlew et al., 2007), this link to plasticity suggests a possible causal relationship. PreNMDARs were also recently implicated in spreading depression (Zhou et al., 2013), a slowly propagating wave of neuronal depolarization that contributes to pathology resulting from stroke and other central nervous system trauma. Here, preNMDARs may sustain spreading depression via regenerative glutamate release (Zhou et al., 2013), although the link to synapse-specific expression *per se* is unknown.

## STP depends on age

Over development, STP switches in several brain regions from high to low probability of release, i.e., from short-term depression to relatively more facilitating short-term dynamics (Bolshakov and Siegelbaum, 1995; Pouzat and Hestrin, 1997; Reyes and Sakmann, 1999; Kumar and Huguenard, 2001; Wasling et al., 2004; Yanagisawa et al., 2004; Zhang, 2004; Frick et al., 2007; Oswald and Reyes, 2008; Cheetham and Fox, 2010; Wang et al., 2012). This developmental change is likely to be due to maturation of calcium signaling and neurotransmitter release mechanisms in the presynaptic terminal (Zucker and Regehr, 2002). As an example, at the calyx of Held, there is a presynaptic change in voltage-dependent calcium channels at around postnatal day 8, from N to P/Q-type calcium channels (Iwasaki and Takahashi, 1998), which is associated with larger postsynaptic responses and less short-term depression at postnatal day 14 (Iwasaki and Takahashi, 2001). Interestingly, the developmental switch of STP occurs later in visual cortex than in barrel cortex, perhaps because the former develops more slowly (Cheetham and Fox, 2010, 2011), which suggests that sensory experience drives this STP switch. This view, however, is not supported by experiments in neocortical organotypic slice (Chen and Buonomano, 2012), where sensory experience clearly is eliminated, yet the STP switch remains. Perhaps activity of any kind, not necessarily driven by experience, is sufficient. However, Wasling et al. (2004) found that the hippocampal developmental switch in STP persisted even when normal activity levels were dramatically reduced in the second postnatal week by tetanus toxin injection. From these two studies, it seems most parsimonious to conclude that an ontogenetic program governs the developmental STP switch rather than experience-driven plasticity. But several studies are in disagreement and are instead supporting the view that experience and activity is critically needed for this developmental maturation of STP (Finnerty et al., 1999; Tang et al., 2007; Cheetham and Fox, 2010, 2011; Takesian et al., 2010; Liu et al., 2012; Takesian et al., 2013). Whether the shift definitely is experience and activity dependent or not in all brain regions and under all circumstances thus remains an open question.

What is the function of this developmental STP switch? Cheetham and Fox (2010) proposed that in early development strong short-term depression of excitation is necessary to prevent runaway excitation since inhibition is not yet fully mature. In adult animals, however, fully developed inhibition may eliminate this need for strong depression, thus permitting excitatory synapses to express a richer spectrum of short-term dynamics. Interestingly, it has also been suggested that this developmental switch might be related to the critical period (Yanagisawa et al., 2004). Although these are quite plausible propositions, the lack of a substantial amount of data obliges us to conclude that the precise function of this developmental switch remains unknown.

One interesting possibility is that the dynamics of different synapse types may mature in a differential manner. In other words, synapse types with similar STP may become dissimilar with age, while those with different synaptic dynamics in the juvenile state may be indistinguishable as the brain reaches maturity. To our knowledge, this possibility has not been extensively explored. One study by Takesian et al. (2013), however, found that thalamic excitation onto fast-spiking INs in gerbil auditory cortex matured toward more short-term depression, whereas the same inputs to low-threshold spiking INs became strikingly facilitating with development. These two types of inputs thus started out with quite similar synaptic dynamics, only to differentiate with age into entirely dissimilar forms of STP. Interestingly, this developmental maturation required activity, since these synapses did not differentiate properly in gerbils with sensorineural hearing loss (Takesian et al., 2013).

## Conclusions and future directions

In this review, we have discussed the functions and mechanisms of STP, with a specific focus on synapse-specific forms of synaptic dynamics. We should point out that, from a naïve and unbiased point of view, there is no *a priori* theoretical reason to believe that STP ought to be specific to synapse type at all—STP could for example be heterogeneously determined by active learning rules that control synaptic dynamics (Markram and Tsodyks, 1996; Sjöström et al., 2003, 2007), or it could just be random; a form of biological noise. As discussed in this review, however, there are numerous examples of synapse-specific forms of STP. Once such synapse specificity of STP has been discovered, it is perhaps less surprising that this specificity can be determined by the presynaptic neuronal type (e.g., Gupta et al., 2000), since neurotransmitter release itself is typically controlled by presynaptic mechanisms (Zucker and Regehr, 2002; Abbott and Regehr, 2004). The existence of target-cell-specific forms of synaptic dynamics, however, may seem surprising and roundabout, since in this case the postsynaptic cell must signal across the synapse to the presynaptic terminal to determine its properties. The fact that such mechanisms do exist (see above and Sylwestrak and Ghosh, 2012) strongly suggests that these target-specific forms of STP are critically important for the proper functioning of the brain.

An emerging principle is thus that STP must be analyzed and understood in the context of the local circuit. This requires combining different scientific disciplines ranging from molecular biology through electrophysiology to computer modeling. This also critically requires that cell types be properly identified, which itself can be a major challenge (Markram et al., 2004; Ascoli et al., 2008). From a theoretical point of view, this suggests that just adding inhibition to a network model of excitatory neurons to balance activity out may sometimes not be enough or even erroneous, since a common theme for all examples of synapse-specific STP is that the most extreme differences are found with respect to different inhibitory IN types. Adding inhibition of one or another type will therefore strongly impact the spatio-temporal structure of network activity. For example, Krishnamurthy et al. (2012) found that the synapse-specific facilitation of excitation onto MCs can drive cortical attractor networks, a role that BCs could not take on because of short-term depression of their excitatory inputs. In general, we believe that the theory of synapse-specific STP deserves more attention, especially since the functional implications can be very difficult to ascertain experimentally.

Still, it is important to recall that STP can also be directly postsynaptically determined, for example due to desensitization of calcium-permeable AMPA receptors (Rozov and Burnashev, 1999; Rozov et al., 2001). Since calcium-permeable AMPA receptors are also synapse-specifically expressed (Rozov et al., 2001; Kullmann and Lamsa, 2007), these receptors may provide a degree of target-cell-specific STP. Another type of postsynaptically derived STP was found in cerebellar stellate cells, which exhibit a gradient of facilitation decreasing from the soma to distal synapses. Distance-dependent facilitation arises from large synaptic conductances depolarizing thin dendrites so much that driving force is reduced, so responses summate sublinearly. This feature potentially makes stellate cells into decorrelators by favoring distributed, non-clustered input activity (Abrahamsson et al., 2012). In addition, postsynaptic temporal summation can itself result in an apparent form of short-term depression, even for connections that are in actuality somewhat facilitating (Banitt et al., 2005).

But we should re-iterate that the postsynaptic neuron does not solely determine synapse type; target-cell-specific STP is merely the specific focus of this particular review article. Indeed, Gupta et al. (2000) summarized their findings in several circuit-organizing principles, the second of which states that “the postsynaptic neuron alone cannot dictate the type of synapse”—accordingly it is still possible for STP to be determined by presynaptic cell type (e.g., Planert et al., 2010). Likewise, plasticity learning rules that impact short-term dynamics (e.g., Markram and Tsodyks, 1996; Sjöström et al., 2003, 2007) are still likely to critically determine computations within a set of synapses of the same type. These three different forms of STP specificity—presynaptic, postsynaptic, or otherwise (e.g., via plasticity)—are thus not mutually exclusive, but can co-exist in the brain. Because synaptic plasticity rules may vary with dendritic location (Sjöström and Häusser, 2006; Kampa et al., 2007; Sjöström et al., 2008; Froemke et al., 2010), this suggests that STP may be regulated on a finer grain than individual postsynaptic cells. Via dendritically local synaptic plasticity rules, STP may in fact be determined by postsynaptic compartment (Branco et al., 2008; Branco and Staras, 2009). Indeed, there is evidence that STP depends on dendritic location (Williams and Stuart, 2002; de Jong et al., 2012), in keeping with this idea, although as discussed above, a similar STP gradient may also arise directly from dendrite biophysics (Abrahamsson et al., 2012).

Furthermore, since most synapses seem to undergo developmental switches in STP, as discussed above, this potentially alters the picture of target-specific STP. For example, connections between PCs in neocortex have been observed to change from depressing to facilitating during development (Reyes and Sakmann, 1999), which would with age potentially render them more dissimilar to those between PCs and BCs (see Figure 1A and Buchanan et al., 2012; Costa et al., 2013). However, a similar developmental change from depression to facilitation has been observed at connections between PCs and putative BCs at low but not high frequencies (Angulo et al., 1999). Some forms of target-specific STP may thus be revealed only by certain activity patterns. And as discussed earlier, Takesian et al. (2013) found that the developmental switch is precisely the opposite for excitation onto fast-spiking compared to low-threshold spiking INs in auditory cortex. These findings thus complicate the picture considerably, which means experimenters must take great care.

The inhibitory circuits activated by target-specific STP are also subject to modulatory and disinhibitory control. For example, monocular deprivation during a critical period leads to a transient reduction in the activity of PV-specific inhibitory circuits in binocular visual cortex, which is permissive for competitive plasticity and ocular dominance shifts (Kuhlman et al., 2013). Similarly, receptive field plasticity in auditory cortex depends on disinhibition mediated by activation of modulatory cholinergic inputs (Froemke et al., 2007), whilst auditory fear conditioning involves cholinergic activation of layer 1 INs which then inhibit L2/3 PV INs (Letzkus et al., 2011). Interestingly, recent evidence suggests that vasoactive intestinal polypeptide (VIP) expressing INs may specialize in such disinhibition, and target primarily SOM and a subset of PV INs (Pi et al., 2013). Because different IN types have distinct forms of STP, a largely unexplored link between synapse-specific STP and regulation of critical period opening and closure thus beckons.

Forms of plasticity other than STP may also be synapse specific and may thus depend on the postsynaptic cell type. For example, long-term plasticity depends on the target inhibitory cell type in neocortex (Lu et al., 2007) and in hippocampus (Nissen et al., 2010; also see McBain and Kauer, 2009). Homeostatic plasticity in neocortex is also dependent on the synapse type (Bartley et al., 2008). These findings further strengthen the principle that synaptic plasticity in general has to be understood in the context of the local circuit.

Another emerging principle is that late-onset INs target the dendrite of local principal neurons, whereas early-onset INs are perisomatically innervating (see Figure 1). This pattern has been found in several neocortical regions (Silberberg and Markram, 2007; Berger et al., 2009), cerebellum (Bao et al., 2010), and hippocampus (Pouille and Scanziani, 2004). In developing visual cortex, this temporal-to-spatial remapping of early BC and late MC spiking across the somato-dendritic axis (Figure 1A) is augmented by preNMDARs that maintain PC-MC neurotransmission during high-frequency firing (see Figure 3 and Buchanan et al., 2012). Recent experiments in awake animals suggest that prominent cortical late-onset inhibition may restrict persistence and spatial spread of activity, thus playing a key role in wakefulness and attention (Haider et al., 2012).

Based on a normative theoretical approach, Pfister et al. (2010) found that STP may make postsynaptic neurons optimal estimators of presynaptic membrane potential. Although the existence of target-specific STP might at first appear to be in contradiction to this theory (see Pfister et al., 2010), another more optimistic interpretation is that different neuronal types may compute different presynaptic statistical properties.

An involvement of target-cell specific STP in disability and disease has also emerged recently. For example, the Fragile X Mental Retardation Protein FMRP regulates neurotransmitter release presynaptically (Deng et al., 2013) and *Fmr1* gene knockout results in pathological STP (Deng et al., 2011b). But the role of presynaptic Fmr1 is specific to target cell type, dramatically affecting STP at synapses onto fast-spiking INs while leaving STP at connections to excitatory cells untouched (Patel et al., 2013). Because of the increased usage of technologies such as 2-photon imaging and paired recordings, the field of synapse-specific STP has become so refined that neuroscientists are now ready to untangle the specific roles of different synapse types in synaptic diseases such as autism, anxiety and epilepsy (Lüscher and Isaac, 2009).

In this paper, we have overviewed the possible functions and mechanisms of target-specific STP. We have listed several examples of STP specific to the target cell, and we have found a few common principles, but it is clear that not nearly enough is known about the why and the how, which means the specificity of STP should be investigated considerably more, both experimentally and theoretically. We argue that to understand the function of target-specific STP, researchers have to examine the results in the context of the local circuit, with its many different cell and synapse types (e.g., Pouille and Scanziani, 2004; Buchanan et al., 2012). Elucidating the role of target-specific STP is therefore likely to require a combination of tools from molecular biology, advanced optics, multiple-cell electrophysiology, and computer modeling.

### Conflict of interest statement

The authors declare that the research was conducted in the absence of any commercial or financial relationships that could be construed as a potential conflict of interest.
